# Questionnaire Measures and Physiological Correlates of Presence: A Systematic Review

**DOI:** 10.3389/fpsyg.2020.00349

**Published:** 2020-03-19

**Authors:** Simone Grassini, Karin Laumann

**Affiliations:** Department of Psychology, Norwegian University of Science and Technology, Trondheim, Norway

**Keywords:** virtual environment, presence, immersion, physiology, review

## Abstract

The published literature has produced several definitions for the sense of presence in a simulated environment, as well as various methods for measuring it. The variety of conceptualizations makes it difficult for researchers to interpret, compare, and evaluate the presence ratings obtained from individual studies. Presence has been measured by employing questionnaires, physiological indices, behavioral feedbacks, and interviews. A systematic literature review was conducted to provide insight into the definitions and measurements of presence in studies from 2002 to 2019, with a focus on questionnaires and physiological measures. The review showed that scholars had introduced various definitions of presence that often originate from different theoretical standpoints and that this has produced a multitude of different questionnaires that aim to measure presence. At the same time, physiological studies that investigate the physiological correlates of the sense of presence have often shown ambiguous results or have not been replicated. Most of the scholars have preferred the use of questionnaires, with Witmer and Singer's Presence Questionnaire being the most prevalent. Among the physiological measures, electroencephalography was the most frequently used. The conclusions of the present review aim to stimulate future structured efforts to standardize the use of the construct of presence, as well as inspire the replication of the findings reported in the published literature.

## Introduction

Presence, which refers to the sense of “being there” in a simulated environment, is a critical concept in the discussion of new technologies and mediated environments (Cummings and Bailenson, 2016). In the academic and industrial communities, there is often an underlying assumption that the main goal for designing virtual environments (VEs, i.e., those environments generated by a computer that simulate some characteristic of reality) is to promote a sense of presence. Cummings and Bailenson (2016) noted that a heightened sense of presence enhances the user's capacity for interaction with the simulation. VEs have found applications in many fields, including clinical therapy, training, learning, and entertainment, due to their capacity to elicit a high degree of presence (Slater and Wilbur, 1997; Nunez and Blake, 2001; Tamborini and Skalski, 2006; Price and Anderson, 2007).

A relatively recent analysis (Cummings and Bailenson, 2016) noted that the systematic investigation of presence as a psychological phenomenon is quite new in the scientific literature. The comprehensive works of Biocca (1997, 1999) are among the earliest scientific efforts aiming to discuss specific characteristics of the sense of presence (such as, e.g., the relationship between mind and embodiment in a virtual medium, the sense of physical presence in a simulated space, and the physical and social sense of presence). Only in the last two decades, starting with the work of Lee (2004), has there been an attempt to provide a more exhaustive explication of presence as a psychological phenomenon by introducing concepts such as social presence (the extent to which other entities presented in the VE “are there” from the user point of view) and self-presence (the sense of the user being able to perceive him/herself as part of the VE).

Researchers have conceptualized the sense of presence in different ways (e.g., Steuer, 1992; Slater and Wilbur, 1997; Witmer and Singer, 1998; Slater, 1999; McMahan, 2003). Furthermore, several terminologies (e.g., telepresence, mediated presence, virtual presence) have been used to refer to the same notion (Lee, 2004). It is also common that the terminologies “presence” and “immersion” are used as synonyms, primarily when used outside the area traditionally covered by psychology (see, e.g., Jennett et al., 2008; Cheng et al., 2015). Generally, the term presence applies to a broad family of phenomena primarily experienced during the use of VE, though this is also reported during the use of different types of displays and immersive media. VE users that experience a high sense of presence often report the feeling of being in a different place than the position in space occupied by their physical body (Slater et al., 1994). Some scholars have proposed that the concept of presence should be treated as a type of perceptual outsourcing or distal attribution (see Loomis, 1992).

It has been a challenge for researchers to develop a widely accepted and unified notion of the phenomenon. Nevertheless, Lee (2004) made a detailed effort, through a comprehensive clarification process, to provide a coherent understanding and a global description of presence (Makransky et al., 2017a). As indicated by the work of Lee (2004), the experiences in real life and those in VEs can be separated into three distinct domains: physical, social, and self. With the term “physical experience,” Lee (2004) refers to the experience of the physical environment and the objects situated in the environment. The social experience domain refers to the experience of other entities with social value in the environment. The self-experience domain is applied to all those experiences that the user has of him/herself.

Furthermore, Lee (2004) hypothesized two different paths by which an experience can be defined as a virtual one. First, an experience can be considered virtual when an artificial technology mediates it. Such artificial technology allows the users to have an experience of a mediated version of the natural, physical space (as in the case of mixed reality). Another VE case is when the human-made equipment enables the experience of environments and entities that do not exist at all in the real world [as in the case of virtual reality (VR)].

Several studies have explored the sense of presence and its possible physiological correlates, but there is a lack of an overview and critical analysis of the various methods. Furthermore, an effort to replicate the findings reported in the literature is missing.

The present review intends to fill the gap through an analysis of the various methodologies used for indexing the sense of presence, with a focus on the questionnaire and physiological methods. Furthermore, it aims to update the results of the comprehensive review on questionnaire use for evaluating the sense of presence published by Hein et al. (2018), as well as previous outdated works (see Insko, 2003) that examined the physiological correlates of the sense of presence. The present investigation asked the following. (1) Which questionnaires and physiological methods have been used together to evaluate the sense of presence? (2) What are the advantages, possible problems, and criticalities of using these methodologies? The answers to these questions will hopefully promote the development of the field as well as the development of a more unified and coherent theoretical research framework for the scientific study of the sense of presence.

### Terminological Clarifications

Defining what presence is and how related concepts can be differentiated from it has posed challenges for researchers. The constructs of immersion, involvement, and emotion are difficult to disentangle from presence itself (Slater, 2003). Several studies have considered the difference between these terms and related constructs (Nacke and Lindley, 2008; Cummings and Bailenson, 2016; Suvajdzc et al., 2018). Generally, immersion refers to the outcome of immersive technology when evaluated objectively (Nilsson et al., 2016). Briefly, a system is more immersive depending on the numbers and quality of delivering displays, in all modalities, and preservation of visual fidelity that is similar to the real world (Cummings and Bailenson, 2016). Technical parameters are related to the level of immersion. Some examples are the field of view, image latency, and frame rate of the image stream (Slater, 1999). On the other hand, presence has been defined as the subjective and psychological reaction to immersive environments (Fromberger et al., 2015). As already introduced in the previous section of the present article, some studies have systematically used the terminology “immersion” and “presence” as synonyms (e.g., Jennett et al., 2008; Amin et al., 2016; Papachristos et al., 2017; Lum et al., 2018). In the present review, the word “presence” will be preferred for the description of the psychological and subjective feeling of being in a VE.

Presence and involvement can be logically separated into two different psychological phenomena (Grabarczyk and Pokropski, 2016). As in the everyday experience, one can be involved in something but not believe that one is present in it (for example, while reading a book or watching a movie; Slater, 2003). Presence involves a variety of emotions, and thus it can be measured by examining the emotional reactions promoted by VE. As noted by Riva et al. (2007), what someone sees may engage him/her emotionally. However, emotional engagement does not qualify as presence *per se*. Various studies have discussed the connection between presence and emotion (Ijsselsteijn et al., 2000; Banos et al., 2008).

Many scholars have used the terms VR and VE synonymously. Although they share similarities, they differ in their context and history (see Luciani and Cadoz, 2007). In the present review, VE will be preferred to describe any immersive visual technology (IVT), while VR will be used to describe those modern IVTs that utilize head-mounted displays (HMD or VR headsets).

### Presence and Human Performance

The importance of studying the sense of presence goes beyond mere scientific curiosity; such study is directly related to applicable research. In this regard, several published studies have revealed a positive relationship between presence and human performance (Baumgartner et al., 2006; Baus and Bouchard, 2017). Questionnaires evaluating the sense of presence (e.g., PQ and SUS) showed it to positively correlate with human performance, and the positive association between questionnaire scores and user performance has been directly used as an argument for a good predictive validity of the questionnaire measures.

As noted by Serafin et al. (2016), presence is essential in VEs because it easily influences the user's behavior, and this factor can be used to modulate the learning performance of users when VEs are used for training. For instance, if the VE is used to train professionals, such as surgeons and firefighters, presence will be vital, since they are expected to perform in the VE similarly to how they will perform in the real world. The study by Stevens and Kincaid (2015) investigated the relationship between performance and presence in virtual simulation training and showed that a high level of presence during virtual training correlates positively with an increase in user performance while performing the trained task in real life.

However, there are some instances in which presence can negatively affect performance (Sharples et al., 2008). A high sense of presence correlates with simulator/cybersickness (Lin et al., 2002), which is a collection of undesirable symptoms often reported by users exposed to VE. However, please note that cybersickness and presence are generally inversely correlated, as reported by a recently published literature review (Weech et al., 2019). Cybersickness negatively affects performance because it triggers disorientation, nausea, and oculomotor symptoms in the user, causing discomfort (Kennedy et al., 1993). Finally, presence may not facilitate every type of performance. For instance, some studies (Mania and Chalmers, 2001; Makransky et al., 2017b) found no association or an inverse correlation between presence and learning outcomes.

Moreover, the sense of presence may not be directly related to task performance: task performance may be more associated with the actual manipulation in the experimental scenario or related to technical characteristics of the human-computer interface (e.g., input lag, visual quality, et cetera). These characteristics, even if connected with the sense of presence (i.e., we can assume that better visual quality generally increases the sense of presence), do not directly represent the subjective sense of presence.

Performance in a VR scenario was directly investigated by a recent study (Rose and Chen, 2018) using different degrees of simulation vividness to modulate subjective presence in the simulation. Nevertheless, the study did not find a relationship between the quality of the simulation (and, consequently, the level of reported presence) and actual (objectively measured) task performance in the VR. Organizations that look to introduce the use of VR into their work routine for training or performance purposes are increasingly interested in studying the possible advantages and disadvantages of the technology at the current stage of research. A recent work (Pallavicini et al., 2019) showed that the visualization of a VE using highly immersive media increases both presence and emotional response. Nevertheless, for some practical applications, especially in highly stressful work scenarios, a high degree of presence and emotional involvement in the simulation may not be a helpful factor, as emotionality may be disadvantageous for training activities or for task performance in VR.

### Measures of Presence

Various studies have attempted to measure presence in a laboratory setting, and four main investigation methods (as noted already by Hein et al., 2018) can be distinguished in the literature: questionnaires, physiological measures, analysis of the user's behavior, and interviews. Baren and Ijsselsteijn (2004) have presented a comprehensive list of the methods (even though outdated). Questionnaires and physiology are two typologies of measures for the assessment of the sense of presence that are often used together. However, several factors (e.g., the many questionnaires used to measure presence and the lack of a standard for the analysis of physiological data), have made a comparison between different studies challenging. The present article tries to analyze the criticalities of using those two methodologies, focusing on the published literature on presence where questionnaires and physiological measures were reported together.

#### Use of Questionnaires

Questionnaires are the most frequently used method for the investigation of presence (see Hein et al., 2018). A standard design employed in studies using questionnaires is to make the experimental participant engage passively or actively in a VE and later ask the participant to answer a survey that evaluates his/her experience. Usually, the questions consist of ordinal scales (e.g., Witmer and Singer, 1998; Lessiter et al., 2001). Some presence questionnaires share similarities: for example, they use a Likert scale that ranges from 1 and 7 [such as the Presence Questionnaire (PQ), Igroup Presence Questionnaire (IPQ), and Slater-Usoh-Steed Questionnaire (SUS)]. Questionnaire instruments have several advantages compared to other methods of investigating presence. They are cheap, easy to administer, and they are applicable regardless of the VE used. Questionnaires do not require lengthy prior preparation, as other methodologies do, and do not require specialized skills or scientific instruments (as physiological measures). On the other hand, the numbers of questionnaires measuring presence, the variety of constructs explored by these questionnaires, and the overall lack of a standard definition for presence as a psychological construct may represent a problem on the use of this methodology. Furthermore, results from studies using different questionnaires may be difficult to compare (Kober and Neuper, 2013). Moreover, questionnaires assessing the degree of presence are susceptible to response bias: where a questionnaire poses queries about presence directly or indirectly, it may possibly load an answer that would not otherwise have reached the participants' conscious level (Szczurowski and Smith, 2017).

#### Use of Physiological Measures

There have been numerous attempts to use human physiological measures as indices for presence (e.g., Meehan et al., 2002; Arndt et al., 2016), and several different types of measures have been identified. These physiological indices can be coarsely divided into two families: brain-related and not brain-related. Among the brain-related measures, electroencephalography (EEG) is one of the most commonly used within the field of cognitive science and has found extensive use in relation to the sense of presence (e.g., Terkildsen and Makransky, 2019). EEG measures the electrical activity of the human brain in a passive and non-invasive way: many neurons disposed perpendicularly to the scalp and firing at the same time produce an electrical potential that is possible to measure from outside the scalp (Breedlove et al., 2010). EEG signals can be analyzed in several different ways. Continuous EEG signals can be divided into frequency bands (usually delta, theta, alpha, beta, and gamma), and those oscillatory neural activities can be interpreted, for example, in connection with human behavior and cognitive processes (Teplan, 2002). Presence has been often investigated using the EEG-based technique of event-related potentials (ERPs; see Kober and Neuper, 2012; Terkildsen and Makransky, 2019). ERPs represent brain activity generated as a response to an event (a stimulation that can be, e.g., visual or auditory). This activity is generally averaged across many samples (trials), in order to reduce signal noise and obtain a reliable estimate for the brain activity related to the response to the stimulation (Andreassi, 2010). The ERP methodology takes advantage of the good time resolution (milliseconds) of the EEG recording and it is widely used in cognitive research (attention, perception, consciousness, etc.). However, EEG has somewhat imprecise signal localization (low spatial resolution). EEG is sometimes also utilized to identify the physical sources of brain signals and connectivity among brain areas (see, e.g., Greicius et al., 2003), and this methodology has also been implemented for the spatial individuation of the physiological correlates of presence (Clemente et al., 2013b).

EEG offers several advantages. It is relatively affordable, even for unspecialized laboratories. The latest developments of portable consumer-grade equipment have made EEG equipment cost-effective and easy to operate. However, the setup of the equipment for the experimental phase is rather lengthy compared with other methods, and data analysis requires specialized expertise. Furthermore, the recording is somewhat sensitive to movements, and data quality could be impaired in VEs where movements are essential. For the latter reason, EEG is not adaptable to all kinds of VEs.

A further brain-related physiological measure that is used for the study of presence is functional magnetic resonance imaging (fMRI; Hoffman et al., 2003). fMRI is a brain imaging technique that allows for spatially precise (millimeters) identification of activity in the human brain. The fMRI scanner can identify the flow of oxygenated blood in the brain in a relatively short amount of time (seconds). This identification occurs due to blood-oxygenation-dependent imaging (BOLD), which highlights the activated brain areas. However, the spatial precision of fMRI comes at the cost of temporal resolution, which is much lower than that of EEG (Huettel et al., 2004). Furthermore, fMRI is very expensive and is generally used for clinical purposes, sometimes only available within hospital infrastructures. The machinery is complex to operate, and specialized medical staff are often required for its correct operation and to limit possible usage risks, further increasing its operational costs. Moreover, there are some non-negligible risks and restrictions for the participants of fMRI studies. For example, a participant cannot participate in fMRI studies if he/she has permanent metal prostheses on his/her body (or, e.g., a pacemaker), as the magnetic field of the scanner may interact with the metal. Eventual presentation of the stimuli (e.g., via VR goggles) needs to be mediated by MRI-scan compatible equipment, i.e., not interacting with magnetic fields. Additionally, the experiment is performed in an unnatural lying-down position, and the head of the subject needs to be immobilized, often causing discomfort. These limitations make this methodology challenging to apply in most, if not all, VEs.

Generally, brain-related physiological measures are more expensive and less adaptable compared to non-brain physiological measures. Several studies have explored the use of galvanic skin response (GSR), also known as electrodermal activity (EDA) or SC [sometimes in the literature as skin conductance responses (SCRs)], which measures how the electrical variations in the skin trigger eccrine sweat glands, a phenomenon that allows SC measurement. In the present article, the abbreviation “SC” will be used to refer to this methodology. The use of SC is well-documented in the literature on human emotion and cognition (Weber et al., 2009; Poels et al., 2012; Chalfoun and Dankoff, 2018). SC is associated, for example, with stress, excitement, engagement, and frustration, and arousal, among other factors (see e.g., Kurniawan et al., 2013).

Furthermore, stimuli that promote attentional processes and attention-demanding tasks relate to several characteristics of the SC signal. SC data can be analyzed in several ways (e.g., decomposing phasic-transient and tonic activity), even though researchers are still in the process of understanding the exact meanings of those different components of SC (see Nagai et al., 2004; Braithwaite et al., 2013; Dawson et al., 2017). SC is easy to set up and minimally invasive, while SC sensors are affordable. However, SC data are sensitive to movements (especially of the part of the body where the sensors are attached) and to all those activities that may modulate the activity of the eccrine sweat glands. Furthermore, SC may not be an optimal proxy for the measure of presence (based on the assumption that arousal/excitement correlates with a higher sense of presence), as SC modulation is highly dependent on the content of the immersive experience. SC can also be significantly modulated by external (often non-controlled) factors, like room temperature or environmental humidity (Boucsein, 2012).

Heart rate (HR) has also been investigated as a possible correlate of presence. In the present review, we do not focus on the various HR analysis methods. However, the most used methods in psychophysiological research are heart rate (HR) and heart-rate variability (HRV). HR is the calculation/estimation of the average heartbeats per timeframe (generally 1 min). HRV, instead, is the measure (in milliseconds) of the changes (i.e., the variability) between successive heartbeats. This time period is called the R-R interval (inter-beat interval; van Ravenswaaij-Arts et al., 1993). The experimental methodology generally preferred for HR studies is electrocardiography (ECG or EKG). ECG records changes in electrical potential associated with the heartbeat (Goldberger, 1998). Due to its affordability compared to ECG, PPG (photoplethysmography) was also used to measure heart rhythm. The PPG can detect blood volume changes in the microvascular tissue, and it is often measured using a pulse oximeter, which illuminates the skin and can detect changes in the light absorption and, with that, indirectly measures the heart rhythm. Heart rate sensors are becoming increasingly cheap, especially PPG sensors. HR is generally less affected by movement than EEG and SC. On the other hand, PPG provides fewer analysis possibilities compared to ECG, giving only the number of heartbeats per time but without giving information on the beat components. However, ECG is more expensive, more challenging to operate, and more invasive than PPG.

Skin temperature (ST) is a rather simple experimental methodology in which a sensor records the temperature of the participant. Even though the use of skin temperature in psychological research date back many years (see Maslach et al., 1972) and has attracted attention in cognitive science research due to its cost-effectiveness and simplicity of use (Lara et al., 2018), this method has not found as many applications as the other methodologies listed above.

Electromyography (EMG) is a technique for recording the electrical activity produced by skeletal muscles. Its setup is easy and inexpensive. However, it finds limited application within the field of cognitive psychology. As subject muscle activity is strongly related to the EMG signal, it has been extensively used in clinical and sports medicine (Steele, 2012). However, due to its connection to subject behavior (muscle movements), it has generally been less used to study cognitive phenomena (with the exclusion of the quite widely used technique of facial electromyography; see Durso et al., 2012). Its robust connection with human behavioral responses makes this technique difficult to use in many VR contexts.

While physiological measures could be more objective indices of the level of presence experienced by a subject, there is no consensus on which measure is the best to use. Furthermore, many of these measures have methodological limitations (e.g., requiring the subject to be still, requiring a long preparation time, and being costly) and therefore cannot be applied for all situations and in all VEs.

#### Behavioral Measures

A third approach for measuring presence is behavioral. It is a sign of a higher level of presence when participants in a VE behave as if they are in a real environment. Some examples of this phenomenon include user behaviors related to conflicting multisensory cues that emanate from both real and simulated environments (Slater, 2002), postural sway (Freeman et al., 2017), and responses to simulated stimuli (Bouchard et al., 2008). Typically, behavioral measures require the introduction of features or tasks into the environment to elicit behavioral responses. For the behavioral approach to be applicable, the VE must present features that trigger either voluntary (e.g., pressing of a button, as in the case of the evaluation of performance; see Slater and Wilbur, 1997), or involuntary (e.g., motion in reaction to an approaching object; see Sanchez-Vives and Slater, 2005) responses. However, these additional tasks or events may be detrimental to the measure of presence. These tasks may not be relevant for the VE, may disrupt the feeling of immersion, or may interact with the VE in uncontrolled ways.

#### Interviews

Hein et al. (2018), in their review, reported that only one study was found that made use of interviews without using a questionnaire. However, even in that case, the presence factors from the interview shared similarities with the typical items of the Presence Questionnaire (PQ, Witmer and Singer, 1998).

## Methods

The methodology utilized here was a systematic literature review of published research articles, following the guidelines stated in the Preferred Reporting Items for Systematic Reviews (PRISMA; see Liberati et al., 2009). The method used for the literature review was based on that reported in the PRISMA checklist. The first step defined the research questions and the setting of the study protocol. Second, relevant research manuscripts were identified, considering only papers authored during the last two decades. Those articles were then screened in different stages, based on their titles, abstracts, and keywords. Afterward, a sub-sample of articles were selected, and their full texts were evaluated. Finally, the information from the reviewed research was summarized and categorized into the topic of interest for the current review.

### Inclusion Criteria and Search Strategy

The inclusion and exclusion criteria were established before the literature review. The inclusion criteria focused on the factors that it was important to evaluate when selecting an article to be part of this review. They limited the articles based on their research design, data, and focus. Research articles that directly investigated the sense of presence were considered for the analysis of measurement approaches to presence. Further criteria were applied to target the articles of interest. First, only papers published from January 2002 to August 2019 were included in order to review the most current literature. This time frame was chosen to not critically overlap with the analysis of a similar review article published in 2003 (Insko, 2003) and to report the current state-of-the-art research. Second, the reviewed manuscripts had to contain an empirical study in which there was some VE experienced by participants. Furthermore, only those studies that reported first-hand experimental data were selected.

The search for relevant papers was conducted using some of the most popular research engines for academic articles: Web of Science, Scopus, and Google Scholar. These scientific databases were chosen for their popularity and for their comprehensive and interdisciplinary nature, which fit the interdisciplinary topic of the present review. Only articles in English were considered for the present review. Only articles published in peer-reviewed journals or conference proceedings were selected during the preliminary screening phase.

During the first stage, the keywords used for the search included “virtual reality” AND “presence,” “virtual reality” AND “immersion,” “virtual environment” AND “presence,” and “virtual environment” AND “immersion.” For Web of Science, the articles in the “Web of Science core collection” database were searched. The queries made by using the “Topic” field, which includes title, abstract, and keywords. Both published articles and conference proceedings were searched for. After the preliminary screening, the results obtained included 3,466 articles. To narrow down the results, the articles were filtered for the categories “Neurosciences,” “Psychology multidisciplinary,” “Psychology applied,” “Psychology experimental,” “Psychology,” and “Psychology biological.” After duplications from every query were eliminated, the number of articles included in the preliminary selection from Web of Science was 371.

For Scopus, the queries were run on the article title, abstract, and keywords of published scientific articles. After the first screening, 6,546 were retrieved. The subject areas “Psychology” and “Neurosciences” were selected to pre-screen those articles. After the filters were applied, 651 results were retrieved.

For Google Scholar, each combination of keywords was queried separately, and the first 10 pages of resulting titles were manually reviewed for each one of the queries. Each page contained 10 results, and the results were sorted by relevance. A total of 140 articles were selected.

The retrieved articles from the three search engines were then imported into Endnote software, and duplicate entries were removed. A total of 875 unique articles were identified. The titles of these articles were screened to distinguish papers fitting the topic of investigation, and 205 articles were selected in the preliminary screening phase. Preliminary screening of keywords, titles, and abstracts was performed to filter the articles further. From this further screening, 120 papers were selected. The full abstract of these articles was then evaluated for inclusion and exclusion criteria, and 59 articles were included for full-text evaluation. Of these articles, only those that used a combination of questionnaires and physiological methods were included. Furthermore, only those articles that clearly attempted to measure presence were selected. Studies that were judged to be clinically oriented and made use of specific subject samples (e.g., people with specific health conditions) were also excluded. Finally, 18 articles were included in the review. The scientist that worked on the selection of the article did not have any conflicts of interest in evaluating the inclusions. However, a degree of subjectivity in choosing the inclusion/exclusion criteria as well as selecting the articles fitting those criteria may have affected the selection of the articles included in the final phase of the review (e.g., the ability of the responsible author, S.G., to understand the methodologies in sufficient detail and evaluate their pertinence to the topic of the present review). This represents a limitation to the systematicity of the present review.

**Figure 1:**
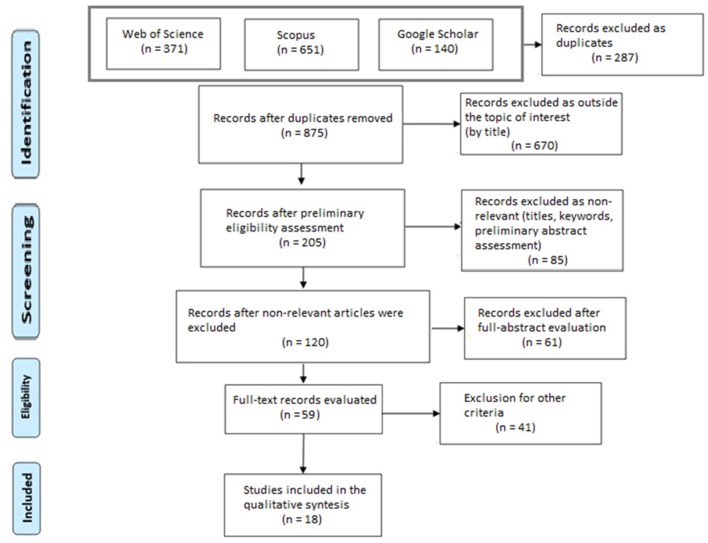
PRISMA flow diagram showing the various stages of article selection.

Table 1 provides a summary of the papers evaluated, including authors and year, measure(s) used for assessing presence, and the main findings reported. Furthermore, only papers that reported at least one physiological measure and one questionnaire instrument for assessing presence were chosen. This design allowed the assessment of the relationship between self-report questionnaires and physiological measures. Articles that only used physiological measures and not questionnaires to assess presence were scarce in the literature (e.g., Bosse et al., 2016). They lacked the subjective self-report component and thus failed to clearly state their criteria for assessing the sense of presence. Finally, twenty articles were included in the main result body of the present literature review and are therefore presented and summarized in Table 1. The first author of the present review (S.G.) had the responsibility for selecting the articles that were included in each stage of selection. However, both authors participated in the discussion for identifying exclusion and inclusion criteria. A PRISMA flow diagram is presented to facilitate understanding of the review procedure (Figure 1).

**Table 1:** Summary of reviewed articles, listed from the oldest to the newest (and alphabetically for publications from the same year).

**Study (Author, Year)**	**Journal**	***N***	**Presence measure used**	**Details/Task**	**Main findings**
Meehan et al. (2002)*	ACM Transaction on Graphics	Multiple experiments, from 92 to 132	Questionnaire (UCLQ) Physiological (HR, SC, ST)	Comparison of participants' physiologicalreactions when in a state of stressful virtual height and a non-threatening virtual room	Presence correlated with change in HR and, to a lesser extent, SC. There were no changes in ST.
Hoffman et al. (2003)*	CyberPsychology & Behavior	7	Questionnaire (scale from 0 to 10) Physiological (fMRI)	VR game that induced low or high presence (visual immersion)	Explored the potential of using fMRI to evaluate the sense of presence. Detailed results were not reported.
Baumgartner et al. (2006)	CyberPsychology & Behavior	23	Questionnaire (MEC-SPQ) Physiological (EEG, SC)	Participants asked to attend a simulation of a roller coaster	Increase in SC responses in the experimental vs. control condition. Event-related power decrease in alpha waves over the brain parietal cortex (cortical activation in the region). Signal localization analyses showed that spatial cues presented during the simulation elicited activity over the parietal cortex and the insula.
Baumgartner et al. (2008)*	Frontiers in Human Neuroscience	77	Questionnaire (MEC-SPQ) Physiological (fMRI)	Subjects presented with a simulation of a first-person roller coaster in different scenarios	Involvement of the right brain and, to a lesser extent, of the left dorsolateral prefrontal cortex (negative correlation with presence) as a neural correlate for presence in adults. Absence of such a mechanism in children.
Busscher et al. (2011)*	Journal of CyberTherapy and Rehabilitation	60 (exp 1) and 44 (exp 2)	Questionnaire (IPQ) Physiological (HR)	Presentation of a neutral virtual world and a virtual flight	HR decreased during the highly immersive VR presentation compared to both neutral presentation and real life.
Kim et al. (2012)*	2012 IEEE Virtual Reality Workshops (VRW)	53	Questionnaire (PQ) Physiological (SC)	Examined the effects of different kinds of VE technologies on human emotions and performance	The most immersive methodologies (CAVE and HMD) produced a higher sense of presence in the users vs. desktop display and increased SC. The CAVE system showed the highest modulation of SC.
Kober et al. (2012)	International Journal of Psychophysiology	30	Questionnaire (PQ, SUS, ITQ) Physiological (EEG)	A spatial navigation task performed using two different presentations: a highly immersive single-wall display (three-dimensional view) and a desktop display (two-dimensional view)	The more immersive media provoke a more intense sense of presence. Task-related power decrease in the alpha band (8–12 Hz) over the parietal cortex correlated with a stronger feeling of presence. A lower reported sense of presence correlated with enhanced brain connectivity between frontal and parietal brain areas.
Kober and Neuper (2012)	International Journal of Human-Computer Studies	52	Questionnaire (SUS, PQ, Short Feedback Questionnaire [SFQ]; Kizony et al., 2006) Physiological (EEG)	Navigation in a virtual city (first-person view)	Auditory event-related potentials (ERPs) elicited by VR-irrelevant tones (oddball paradigm) were indexes of presence experience. The reported increase in the sense of presence correlated with the allocation of attentional resources toward the simulated environment (as opposed to the real environment). Late negative slow waves were the best indicators for presence experience in VR, but not mismatch negativity (MMN) or earlier ERP components.
Poels et al. (2012)	Cyberpsychology, Behavior, and Social Networking	19	Questionnaire (PQ) Physiological (SC, EMG)	Investigated the emotion felt by gamers during a videogame-playing session	The measured level of arousal and subjective pleasure were good predictors of the user's sense of presence. SC and electromyography were used as an estimator for arousal.
Clemente et al. (2013a)	Interacting with Computers	14	Questionnaire (SUS) Physiological (fMRI)	Navigation task in a VE consisting of a clean bedroom with basic furniture and accessories	Several brain regions increase their activity during navigation in the VEs (frontal, parietal, and occipital). Activity in the dorsolateral prefrontal cortex was reduced during the navigation task. The centro-parietal cortex and insula were positively modulated by the navigation task; increased activation was related to the sense of presence.
Clemente et al. (2013b)	Studies in Health Technology Informatics	10	Questionnaire (SUS) Physiological (EEG)	Navigation task in a VE consisting of a virtual bedroom containing some standard items (bed, closet, desk, etc.)	Insula activation was correlated with the sense of presence. This brain area may be related to stimulus attentional allocation and self-awareness processes. Portable EMOTIV EPOC EEG was used.
Burns and Fairclough (2015)*	International Journal of Human-Computer Studies	20	Questionnaire (IEQ) Physiological (EEG)	Playing a computer game via two different visualization methods; modulation of sense of presence using different stimulus-presentation devices	The technology of visualization of the video game utilized did not affect the EEG signals recorded (ERPs). They used a dual-oddball task as in Kober and Neuper (2012). As in the latter study, the late ERP component was modulated by task involvement.
Anderson et al. (2017)*	Aerospace Medicine and Human Performance	18	Questionnaire (Modified Reality Judgment and Presence Questionnaire [MRJPQ]) Physiological (SC, HR)	Participants were asked to passively view a VE	Reductions in SC from baseline were greater at the end of the natural scenes compared to the control scenes. HR results were inconclusive.
Lee et al. (2017)*	IEEE 2017: Virtual Reality	41	Questionnaire (PQ, Social Presence Questionnaire; Bailenson et al., 2003) Physiological (SC)	Participants were asked to rate their sense of presence when experiencing various degrees of multi-sensory stimulation: “mute,” “vibration,” and “sound.”	Increase specifically in the sense of “social presence” in the “vibration” condition compared to the “sound” condition. Increase in general sense of presence both in “vibration” and “sound” conditions compared to a mute presentation. SC was not different among conditions.
Makransky et al. (2017b)*	Learning and Instruction	52 (two groups of 28 and 24)	Questionnaire (rating 1 to 5) Physiological (EEG)	A teaching scene (science simulation) was presented using a traditional desktop display (PC) or a VR headset	The VR condition increased the sense of presence, but the learning effect was lower compared to the display presentation. Cognitive load (EEG activity based on the calculation reported by Berka et al., 2007) was higher in the VR condition.
Gromer et al. (2019)*	Frontiers in Psychology	49	Questionnaire (MEC-SPQ) Physiological (SC, HR)	Immersive simulation of height; participants were selected for being sensitive to height but not clinically phobic	Experiencing emotions in a VE gave a stronger feeling of being there. The emotional factors were detected by physiological measures.
Pallavicini et al. (2019)*	Simulation & Gaming	24	Questionnaire (SUS) Physiological (SC, HR)	Participants played a game (first-person shooter) using different visualization technologies to induce a lower or higher degree of immersion (desktop monitor vs. Oculus Gear VR)	No difference in “game difficulty” between desktop video and VR. The study participants showed enhanced emotional responses in VR. The reported sense of presence was also higher in the VR condition.
Terkildsen and Makransky (2019)	International Journal of Human-Computer Studies	34	Questionnaire (MPS) Physiological (SC, EEG)	Participants explored and interacted in the scenario of a game	In MMN, the N1 components were presence indicators. Skin response peaks/minimums were also found to be presence indicators.

The names of the questionnaires, as well as the names of the physiological measures utilized, are abbreviated; please consult the Appendix. Articles indicated with ^*^ made use of head-mounted displays or similar devices.

## Results

The study results are presented in the following paragraphs. They are firstly analyzed regarding the questionnaires used, and in the following section of the review, regarding the physiological measures. A critical overview of the measures and the main findings of the analyzed articles is also presented.

### Questionnaires

The most utilized method for evaluating presence is by self-reporting questionnaire (Hein et al., 2018). According to the articles selected for the current review, the most commonly used questionnaires were the PQ, SUS, ITQ, and MEC Spatial Presence Questionnaire (MEC-SPQ). The PQ was by far the most frequently used self-report measure in the analyzed literature, a result that confirms the findings of the literature review by Hein et al. (2018). Schubert et al. (2001) already noted a few years after its introduction that the PQ questionnaire was widely used in a variety of different fields, including studies on general presence, social presence, simulations, storytelling, and games. The SUS Questionnaire was the second most used questionnaire in the body of literature analyzed.

Other studies used less conventional questionnaires, such as the Modified Reality Judgment and Presence Questionnaire (MRJPQ; Anderson et al., 2017), IPQ (Schubert et al., 2001), University College of London Questionnaire (UCLQ; Usoh et al., 1999; Meehan, 2001), and Multimodal Presence Scale (MPS; Makransky et al., 2017a). Several studies preferred simpler scales (e.g., Hoffman et al., 2003), constituted by a single item that queried the general sense of presence. Some studies used a combination of questionnaires, and the results from different measures of presence correlated, as was the case for the PQ and SUS (Salanitri et al., 2016; Skarbez et al., 2017). One of the studies analyzed (Lee et al., 2017) employed a specific questionnaire (in addition to the PQ) that aimed to evaluate social presence (Social Presence Questionnaire, Bailenson et al., 2003). However, the results of the two questionnaires were not directly compared.

There are many other questionnaires that have had some resonance in the literature but were not presented in the current literature review because they are not associated with physiological measures in the published research body. For example, the E^2^I Questionnaire has been adopted in studies that emphasized the role that enjoyment has in presence (Lin et al., 2002). Following the same rationale, Frommel et al. (2017) explored enjoyment as a vital part of the VE, including enjoyment items in a modified version of the PQ questionnaire. Similarly, there are other studies that considered more specific measures of what is referred to as “spatial presence” (MEC-SPQ; Vorderer et al., 2004). The sense of “spatial presence” was specifically defined as the feeling of being present within the physical body, as well as feeling able to interact in the VE (Lee, 2004). Sohre et al. (2017) also used a customized questionnaire to evaluate attention (and, specifically, spatial presence). Witmer and Singer (1998) published the Immersive Tendencies Questionnaire (ITQ), which is a modification of the PQ they had designed earlier. The researchers used this questionnaire in combination with the PQ in several studies (Witmer and Singer, 1998; Skarbez et al., 2017).

Overall, the published research evidenced the use of a wide variety of questionnaires in association with physiological measures. Furthermore, when their items are more closely analyzed, these questionnaires were found to differ significantly in terms of scope and details. In principle, the questionnaires aim to quantify the same construct (presence), but intrinsic differences exist due to the designer's conceptualizations of presence, as noted by Slater (1999) in his critique of Witmer and Singers' PQ. Of the questionnaires presented in the reviewed literature, the majority aims to explore the concept of “physical presence” (PQ, SUS, IPQ, and MRJQ); however, the way physical presence is described varies among the instruments. The PQ questionnaire emphasizes the “involvement” and “immersion” characteristics of the simulated environment, while the SUS and IPQ are focused on the sense of “being there” (i.e., the sense that the experienced VE may be part of the reality). The MEC-SPQ questionnaire analyzes what is called “spatial presence,” specifically in the framework of the MEC Two-Level Model of Spatial Presence (Vorderer et al., 2003). The latter model theorizes a clear separation between the constructs of presence, involvement, and attention, and factors such as involvement, self-location, and the possibility of performing real action in the VE. Other questionnaires are less clear about the theoretical model they follow. For instance, the Immersive Experience Questionnaire (IEQ) does not directly discuss a model in its theoretical formulation but elaborates more generally about theories of cognitive involvement, absorption, and flow (Jennett et al., 2008). In contrast, the MPS questionnaire clearly states its theoretical starting point as Lee's (2004) theory, as well as the types of presence assessed in its inventory (physical, social, and self-presence). A detailed overview of the questionnaires reviewed in the present review is given in Table 2.

**Table 2:** Summary of the characteristics of the most utilized questionnaires in the analyzed articles.

**Name**	**Number of items**	**Explored concept**	**Theory/formulation**	**Subscales**	**Factors/Indicators/ Dimensions**	**Reliability**	**Construct validity**	**Predictive validity**
PQ	19	Physical presence	The sense of involvement and immersion are different, and both are necessary for developing the sense of presence. Involvement: focusing attention and energy on VE. Immersion: self-perceiving as a part of the VE stimulation.	Involvement/control, Natural, Auditory, Haptic, Resolution, Interface quality	Control, Sensory, Distractor, Realism	Cronbach's α = 0.88	Items based on the factors derived from a review of the literature. PQ items investigate presence as involvement and immersion. Positively related to ITQ (*r* = 0.24).	Positively related to task performance. Negatively related to simulator sickness. Validity was inconclusive when testing for performance (Youngblut and Huie, 2003).
SUS	6	Physical presence	Presence is treated as a mental state. Users are physically present in the VEs (from the formulation of Draper et al., 1998).	None	Being there, VE as more real or present than reality, Locality (VE as a visited place)	Not reported	Not reported by the author of the questionnaire. Positively related to PQ (*r* = 0.51).	Negatively related to the number of errors in the VE task. Validity was inconclusive when testing for performance (Youngblut and Huie, 2003).
MEC-SPQ	3 versions: 4, 6, or 8 items per subscale	Spatial presence	The MEC Two-Level Model of Spatial Presence (Vorderer et al., 2003); distinction among presence, involvement, and attention.	Attention Allocation, Spatial Situation Model, Spatial Presence: Self Location, Spatial Presence: Possible Actions, Suspension of Disbelief, Higher Cognitive Involvement, Domain Specific Interest, and Visual-Spatial Imagery	Spatial Presence: Self Location, Spatial Presence: Possible Actions, Cognitive Involvement	Assessed separately for each scale, Cronbach's α from 0.78 to 0.94.	Not reported	Not reported by the author. Positively correlated with user performance (Morrison et al., 2009)
IPQ	14 (using items from the SUS and PQ)	Physical presence	The sense of presence emerges from the creation of a spatial-functional mental model of the VE. Cognitive processes related to this model are(1) construction of the representation of body action as real possible actions within the VE and (2) suppression of external sensory inputs that are incompatible with the VE.	Spatial Presence, Involvement, Realness	Transportation, presence as immersion in the VE; presence as the “realism” of the VE	Cronbach's α = 0.85 and 0.87 (from two preliminary studies)	Data collected using this questionnaire showed a similar factor structure compared to other questionnaires. Confirmatory factor analysis of the model that consists of three factors confirmed the validity of the measure (Schubert et al., 2001).	Ability to distinguish multiple levels of presence (van Schaik et al., 2004; Riecke and Schulte-Pelkum, 2015)
ITQ	18	Involvement	The items assess the tendency to become involved in activities and the ability to focus on one specific activity. Investigates both involvement and immersion.	Not reported	Focus, involvement, tendency to play games	Cronbach's α = 0.75	Based on the items from the analysis of previous literature. Correlates with PQ.	Not reported
IEQ	31	Mixed: physical presence and theories of cognitive involvement	Built on theories of flow, cognitive absorption, and presence. Five dimensions of cognitive absorption (Agarwal and Karahanna, 2000). Operationalized based on available items from the literature.	Basic attention, temporal dissociation, transportation, challenge, emotional involvement, enjoyment	Cognitive Involvement, Real World Dissociation, Challenge, Emotional Involvement, Control	Not reported	Factor analysis (principal component analysis)	Not reported
MRJPQ	15	Physical presence	Items build to evaluate the extent to which the user feels physically in the virtual world and an emotional impact from the simulated scenario	Not reported for the modified version	Not reported for the modified version	Cronbach's α = 0.88	Not reported for the modified version.	Not reported for the modified version.
MPS	15	Mixed: physical, social, and self-presence	Lee's (2004) theory of presence, division of presence into three sub-dimensions: physical, social, and self.	Physical presence, Social Presence, Self-Presence	Physical realism, not paying attention to the real environment, sense of being in the VE, not aware of the physical mediation, sense of coexistence, human realism, not aware of the artificiality of social interaction, not aware of the social mediation, sense of bodily connectivity, the sense of bodily extension	Good reliability measured in different experiments for each of the subscales in different studies (using the Person separation index and Cronbach's alpha)	Based on the items from the analysis of previous literature. The scale was validated using confirmatory factor analysis and item response theory.	Not reported by the authors. Physiological index of attentional allocation (Terkildsen and Makransky, 2019).

*This overview is based on the original publication of the scales and published literature. Please see the Appendix for full questionnaire names*.

With the proliferation of technologies that aim to simulate interactions between people and environments, there is a need to evaluate presence metrics to build measurements that can consistently predict the sense of presence of the users (based on a unified theory). Thus, developing questionnaires to measure presence properly is still an ongoing scientific endeavor. Factor analysis was used in the analyzed articles to test the construct validity of the MPS, IPQ, and IEQ specifically reported in that article. The predictive validity of the questionnaires is sometimes stated in the original article. As reported in Table 2, this factor was most commonly assessed by measuring the positive correlation with various types of task performance (PQ and SUS). However, the later work of Youngblut and Huie (2003) found inconclusive evidence for the ability of those questionnaires to predict task performance in VEs.

Items for many of the analyzed questionnaires were sometimes unclear or difficult to grasp for a naïve participant, e.g., “How is your memory of the scenario similar to being in real places?” (SUS; Slater and Steed, 2000) or “How compelling was your sense of objects moving throughout space?” (PQ; Witmer and Singer, 1998). A recent effort to build a unitary scale for the measurement of various aspects of presence was made with the development of the MPS (Makransky et al., 2017a). This effort represents a step toward a standard and widely accepted measure of presence for various types of VEs. The scale attempts to assess the three major senses of presence in virtual environments (Lee, 2004): physical presence (like several previously reported scales, including PQ and SUS), social presence, and self-presence. Another major advantage of the MPS scale is its clear theoretical standpoint (Lee, 2004), which has been validated by multiple experiments according to tests performed using modern test theories (i.e., confirmatory factor analysis, as described in Brown, 2014) and the Item Response Theory (as described in Embretson and Reise, 2013).

**Figure 2:**
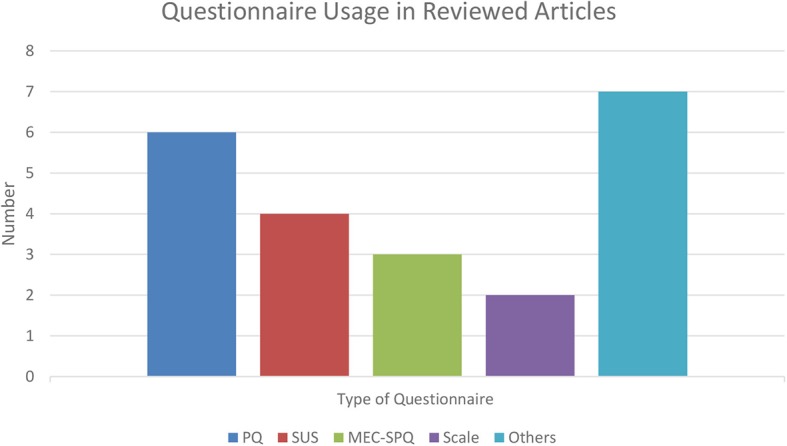
Usage of presence questionnaires in the reviewed articles. See the Appendix for full questionnaire names.

It is worth noting that the current review may not adequately represent the entire literature published in the last two decades, as several factors may have influenced the selection criteria of the sample of articles included. Therefore, the prominence of the measures of presence that were distinguished may not be generalized outside the sample of articles analyzed here. However, our findings (e.g., the PQ as the most-used questionnaire) are coherent with previously published reviews (see Hein et al., 2018). Figure 2 presents an overview of the prevalence of questionnaires used in association with physiological measures.

### Physiological Measures

Psychophysiological studies use a variety of instruments to evaluate the relationship between psychological and physical states in both experimental and naturalistic settings. In tandem with modern brain image methods, psychophysiological approaches have enhanced the understanding of the interplay between physiological indices and several human cognitive processes.

Numerous studies aimed to find physiological measures that correlate with the self-reported sense of presence, using a variety of methodologies. The published research shows a variety of physiological measures that correlate with subjective self-report of presence. EEG was commonly analyzed using both spectral signal decomposition and ERPs. The ERP paradigms commonly assessed presence indirectly, specifically by studying the attentional processes allocated toward the simulated vs. the “external” real environment. In order to do so, the researchers used a traditional auditory dual-task oddball paradigm. In this paradigm, a series of frequent, repeated auditory tones are followed by less frequent tones of a different pitch, a phenomenon that generates a cognitive mismatch in the listener. This experimental design is often used in the study of subjective awareness, in combination with EEG (Näätänen et al., 2019). Burns and Fairclough (2015) used the Kober and Neuper (2012) paradigm in their study and found that task difficulty significantly increases immersion. They also replicated the connection between slow-wave amplitude in response to oddball auditory stimuli and sense of presence. However, the recent work of Terkildsen and Makransky (2019), who employed a very similar study design to both Kober and Neuper (2012) and Burns and Fairclough (2015), did not replicate their findings. Instead, these authors reported an earlier ERP component correlated with presence (N1 and MMN). Clemente et al. (2014) used EEG to measure presence for navigation in virtual environments, using consumer-grade EEG equipment (Emotive EPOC EEG).

Only a limited number of fMRI studies (three) were found in the literature (one of which did not report detailed findings; Hoffman et al., 2003), reporting only partially coherent results.

Among the non-brain physiological indices, presence was found to positively correlate with SC activity (Baumgartner et al., 2006; Kim et al., 2012), but Meehan et al. (2002) reported only a weak association and suggested that HR is a better correlate for presence. Furthermore, Terkildsen and Makransky (2019) found that the sense of presence may differently modulate different SC components: SC peaks/minima are good predictors of the sense of presence, while SC response magnitude shows no association with it. Another study (Lee et al., 2017) found no association between presence and SC (using the sum of skin responses for each of the three conditions employed in their experiment).

HR was found to be one of the most reliable indices of presence among the physiological indices, and it can be compared directly with other physiological measures (Meehan et al., 2002), except in the case of the study of Busscher et al. (2011), which found negative correlation between HR and presence in a simulated flight scenario. The authors of the latter article proposed that the average lower heart rate during the virtual flight (compared to a neutral VE condition), could reflect participant coping mechanisms. A similar reduction in HR has been observed in other studies presenting stressful stimuli (Bosch et al., 2001; Busscher et al., 2010). Furthermore, analysis of HR variability (Anderson et al., 2017) was shown not to be a reliable index of the sense of presence. Some studies analyzed in this review (e.g., Pallavicini et al., 2019) explicitly did not use HR to study presence *per se*, but rather, they used it to index connected factors, such as emotional responses.

**Figure 3:**
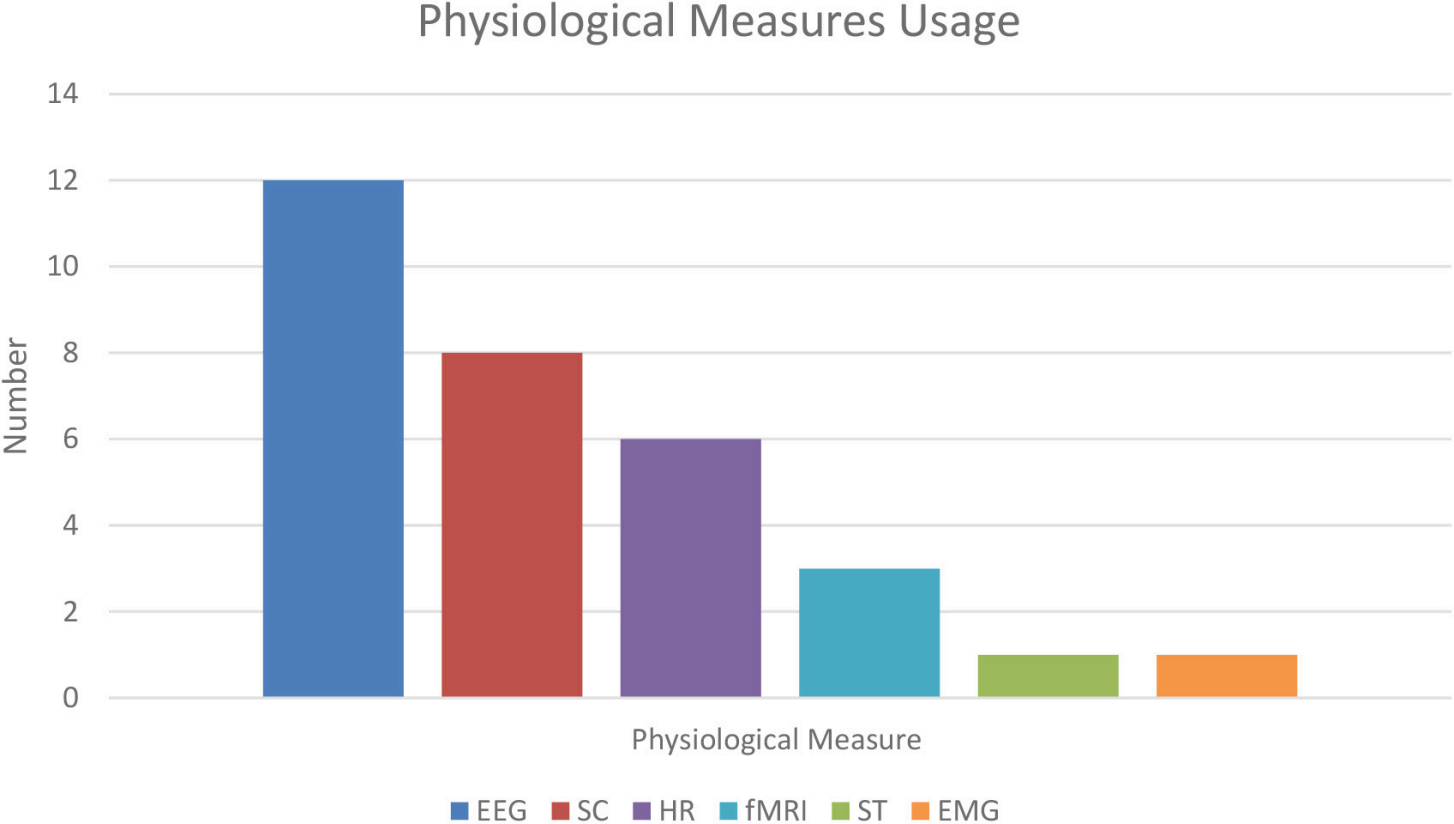
Physiological measures in the reviewed studies. SC, skin conductance; EEG, electroencephalogram; HR, heart rate; fMRI, functional magnetic resonance imaging; ST, skin temperature; EMG, electromyography. As noted from the chart, EEG is the most commonly used physiological measure, followed by SC.

One study investigated ST as a possible index of presence (Meehan et al., 2002); even though the authors expected skin temperature to be an index for enhanced presence in their more highly arousing experimental condition (according to previous investigations using sense of height as exposure; see Andreassi, 2010), there was no connection between the phenomena. Unfortunately, the use of ST as a possible index of presence was not found in other articles included in the present review. More studies are needed to gain a better understanding of ST as an index for presence. One study also combined EMG with SC to assess the sense of presence (Poels et al., 2012). The results of the EMG confirmed those from SC. However, in the context of the study of Poels et al. (2012), EMG was used as a measure of arousal and not of presence. Unfortunately, such a finding was not replicated nor attempted by other studies, and therefore it is challenging to give an interpretation to it. For an overview of the number of uses of physiological measures in the reviewed articles, see Figure 3. The prevalence of the psychophysiological indices reported in the present review may be characteristic of the sample of studies retrieved and may be difficult to generalize when also considering studies that have been excluded from the current review.

## Discussion

In the present review, articles from the last two decades that examined both questionnaires and physiological measures for the assessment of the sense of presence in VEs were analyzed. The PQ was the most often-used instrument. This review showed that a wide range of questionnaires had been developed and utilized for assessing presence. Furthermore, it was noted that those questionnaires, even if they aimed to measure the same constructs, are rooted in several different theorizations of presence. Indeed, they have a very different number of items and use a variety of distinct sub-scales. These scales, however, seem to generally correlate with one another (e.g., PQ with SUS and PQ with ITQ). Construct validity of the scales was generally assessed by reviewing the already existing literature; sometimes, factor analysis (IEQ or MPS) was utilized to assess it. Human performance was used directly to validate the effectiveness of the most-used scales (but see Youngblut and Huie, 2003).

A wide range of physiological measures was used together with questionnaires, attempting to identify physiological correlates of the sense of presence. The most prevalent measure in the retrieved literature was EEG. The attempts to use SC and HR or to exploit other cognitive phenomena during EEG recordings to assess the sense of presence have revealed mixed results. Even though many studies explored the sense of presence, many criticalities were identified in the present literature review. The study of presence is made complex by the degree of subjectivity of the construct, the different modalities of inducing presence (and their evolution over the years), and the difficulties related to the communication of the feeling of presence (Slater, 2004; Sanchez-Vives and Slater, 2005; Oh and Rosakranse, 2014; Cummings and Bailenson, 2016).

Presence is the sensation of being in the place presented in a VE (Nash et al., 2000). It is characterized by the illusion that the virtual events are real, and it is fundamental to determine the extent of presence in those simulated environments (Slater et al., 1994). The “illusion” or “feeling” of presence is highly subjective, and there is no developed and generally accepted method for its evaluation. As shown in the present review, researchers have used markedly different means to measure the phenomenon, such as questionnaires featuring widely different items and a variety of physiological responses. Furthermore, findings from the physiological responses were obtained using a variety of methodologies and diverse phenomena within the same methodology (e.g., brain wave oscillation analysis vs. event-related potentials; peak amplitude analysis vs. overall skin conductance).

Measuring presence using questionnaires is justified by the fact that it is a subjective concept (Nichols et al., 2000). As such, questionnaires are commonly considered the most appropriate measure for a person's subjective experience. Previous studies demonstrated that experiments for evaluating presence using questionnaires employ manipulative aspects of VE. In most cases, the responses are based on Likert scales (Nichols et al., 2000; Lessiter et al., 2001; Slater et al., 2010). As noted in the study by Lessiter et al. (2001), a variety of questionnaires are utilized, and they are often based on different definitions of presence. The present review attempted to clarify and summarize the characteristics of the most used questionnaires (see Table 2). As already evidenced in the Results section, those questionnaires utilized many different theorizations of the construct of presence, for example, the theory of multidimensionality of presence of Lee (2004) employed by the MPS, and the theories of cognitive flow (see, e.g., Agarwal and Karahanna, 2000) used by the MRJPQ. This has created an astonishing number of measures for presence; see the criticism of the proliferation of questionnaires assessing presence by Nordin et al. (2014) and the comprehensive (but outdated) list of presence questionnaires presented by Baren and Ijsselsteijn (2004).

There are several benefits with psychophysiological measures, in case reliable ways to measure the sense of presence with only physiological measures are identified. These benefits include the possibility of continuous data collection and, therefore, the ability to study the association of physiological activity with the effect of a contemporary stimulus presentation in experimental paradigms. Psychophysiology can also help in the investigation of phenomena that are difficult to capture using self-report methods. Thus, this modality will provide a more accurate analysis of the physiological state of a person compared to questionnaires or bare behavior.

Consumer-oriented, cost-effective, small, and easy to handle EEG systems are now available and have already been used for the study of the sense of presence (Clemente et al., 2013b; Tarrant et al., 2018). However, even though validation against medical- or research-grade equipment has been undertaken (see, e.g., Badcock et al., 2013), these consumer-oriented EEGs have been shown to record a less reliable signal compared to their—generally more expensive—professional counterparts (see Duvinage et al., 2012, 2013). Therefore, results from studies that utilize these tools may generally be more inaccurate and difficult to replicate. Furthermore, EEG (with regards to the study of presence) is often used by employing very different experimental paradigms and methodologies that are impossible to compare directly. Additionally, as in the case of the recent study of Terkildsen and Makransky (2019), some published results are difficult to replicate, even when employing almost identical paradigms. However, it is worth noting that Terkildsen and Makransky (2019) used the MPQ to assess subjectively reported presence, a measure that shows essential differences compared to the questionnaire used in previous studies that employed the same paradigm (SUS and PQ in Kober and Neuper, 2012; IEQ in Burns and Fairclough, 2015). On the positive side, Tauscher et al. (2019) studied the methodological feasibility and analyzed the possibility of using EEG equipment combined with VR headsets. They validated the possibility of combining these methods and reported a relatively small and predictable interference (easy to clean) from the VR headset with the recorded signal. As Millán et al. (2010) noted, EEG is widely implemented in the development of brain-computer interface (BCI), and therefore it is expected that in the next decades the use of this methodology will grow exponentially in the tech sector and possibly in the study of brain activity during immersive experiences.

The few fMRI studies reported in the literature appear to generally agree on the involvement (negative correlation) of the dorsolateral prefrontal cortex in the experience of the sense of presence and on the role of the insula. However, Clemente et al. (2013a) reported the inactivation of the dorsolateral prefrontal cortex in a different, inferior location compared to Baumgartner et al. (2008). Related brain areas were also shown to activate in EEG studies (Clemente et al., 2013b). However, the source localization attempt carried out in the EEG study of Clemente et al. (2013b) was conducted with a sub-optimal configuration: the researchers used data from low-density (14-channel) consumer-oriented EEG equipment to perform signal source-localization analysis (see Staljanssens et al., 2017; Michel and Brunet, 2019) and with a small participant sample (10). Further studies are needed for a more precise spatial localization of the phenomenon and to confirm it. The activation of the insula is related to cognition and behavior, as for emotion, regulation of the body's homeostasis, perception, motor control of hands and eyes, self-awareness, cognitive functions, and interpersonal experience (Karnath et al., 2005; Craig, 2009; Clemente et al., 2014). Self-awareness, sense of agency, and sense of body ownership are essential in this context, as directly linked with the sense of presence. Attentional and behavioral components are crucial for the development of the sense of presence, such as increasing the ability to understand the dynamics, predict, and interact with the VE (Sjölie, 2012). Clemente et al. (2013a) acknowledged that their interpretation of the data was speculative and that the brain areas activated during their VE navigation task are possibly not directly related to the sense of presence *per se*.

The reviewed literature showed that little effort had been made to replicate the physiological indices proposed for the feeling of presence, despite the ambiguous results sometimes reported and the variety of different methods used for indexing the phenomenon. When replication was deliberately attempted, the early results were not always successful (Terkildsen and Makransky, 2019). SC and HR studies generally showed more consistent findings, with a higher level of immersion correlating with greater SC or increased HR. However, Busscher et al. (2011) reported a negative correlation between HR and presence and proposed this to be a result of the “conservation-withdrawal” (see Bosch et al., 2003) coping response to a more arousing VE experience. If this interpretation is correct, HR may be very sensitive to the content of the simulation, and its use for the study of presence may be impaired.

While fMRI and EEG are more likely, in the future, to pinpoint specific brain-related activity patterns or areas directly involved (or highly correlated) with the sense of presence (as in the somewhat related field of awareness; see Koivisto and Grassini, 2016; Koivisto et al., 2016, 2017; Jimenez et al., 2018), SC and HR are more likely to identify secondary effects of presence, as well as experiences modulated by or together with presence, such as, for example, arousal, emotion, and stress (Poels et al., 2012; Chalfoun and Dankoff, 2018). Furthermore, there are myriad data collection and data analysis modalities for SC (SCR, SCL, and GSR peak amplitude/number, just to cite a few), and this makes the comparison of the various methodologies that employ SC almost impossible.

Methodological problems in the study of presence include over-reliance on the use of the self-evaluating questionnaires and the difficulty in understanding and elaborating the questionnaire items for the naïve participants involved in the experiments. Additionally, questionnaire methods only allow presence in VEs to be evaluated in a non-invasive manner and only after the experience, while attempting to do it during the experience comes at the cost of breaking the participant's sense of presence. Thus, an approach to indirectly measure presence via, for instance, non-invasive and reliable physiological measurements, would be ideal for understanding the ongoing presence level of the users without the need to query them about their subjective feelings and interrupting their immersive experience to collect data.

Physiological measures may provide numerous advantages compared to other types of measures in the future, for example, continuous, non-invasive, real-time, and relatively objective assessment (Kavikanga et al., 2011). Unfortunately, these measures are difficult to use alone in the current state of research due to the lack of verified and easily replicable physiological indices of presence that are highly correlated with self-reports. Furthermore, several problems are present in the use of physiological measures compared to questionnaire instruments to assess presence. Recording equipment is very sensitive to motion (EEG, SC, and HR), and, in fMRI, motion by the subject is not allowed at all due to technical constraints. Therefore, not all experimental scenarios will be suitable for those methods.

Moreover, VE content can affect the recorded data and thus constitute an uncontrollable variable when comparing among different experimental settings. It may be challenging to isolate the phenomena of “presence” *per se*, as many other cognitive or perceptive factors (e.g., emotional charge of the environment, arousal of the subject, and image quality) may profoundly influence the physiological data. Only systematic replicative efforts of published studies, as well as future investigations that aim to isolate the phenomenon of presence and reduce the influence of confounding factors, can help to identify reliable physiological correlates of presence. An optimal physiological correlate of presence should be able to discriminate the level of the user's presence with a high correlation with self-reported questionnaires and independently from the content of the VE experience.

Immersive media are currently used as a popular source of entertainment (Williams and Mascioni, 2017). It is, therefore, important to comprehensively evaluate the user experience, and the sense of presence is often used as an index of quality for virtual environments. Additionally, understanding and measuring presence has become necessary due to the various applications of virtual reality in different fields, as noted by Schuemie et al. (2001). Furthermore, several studies demonstrated that presence could be a crucial factor to consider when using VE outside the entertainment context, for example, as a training tool in work environments or to increase the performance of users (Baumgartner et al., 2006; Baus and Bouchard, 2017).

## Conclusions

The present review analyzed the body of scientific literature on the measurement of the sense of presence published during the last two decades. The analyzed studies were selected from those reporting both physiological and questionnaire data. In the introduction of the present review, emphasis was placed on clarifying the most common definitions and propositions for presence and related concepts. Furthermore, the review replicated and updated the results of the comprehensive review on questionnaires used for evaluating the sense of presence published by Hein et al. (2018), as well as previous work (Insko, 2003) that examined the physiological correlates of the sense of presence.

Overall, there was no standard measurement method for presence, even though there is a growing body of literature that compares various measurement constructs. One of the most important findings reported in this review is the reliance on the use of questionnaires (and the diversity of questionnaires). Considering that in the coming years, VR technology users will probably increase in number, there is a need for research on standard practices and standardization in the area to understand the effect of those technologies on the end-user, as well as to help in the development of better ones in the future.

At the current state of research, no physiological measure has collected enough evidence to be considered “good enough” to be reliably used alone, without the user giving their subjective evaluation of the experience. The nowadays quite outdated study of Meehan et al. (2002) compared several non-brain physiological measures and showed that HR may be a better measure than SC and ST. Other studies have also shown the generally acceptable reliability of HR for evaluating presence. However, the measure was found not to be reliable when HR variability analysis was attempted (Anderson et al., 2017) and was shown to be possibly too sensitive to the content of the VE to be successfully used in emotional/arousing VE scenarios (Busscher et al., 2011). A combination of different types of measures (e.g., questionnaire, behavioral, and physiological), may be, in the current state, the better approach for properly evaluating the sense of presence in a VE.

Physiological measures, especially SC and EEG, showed widespread use. However, despite contrasting results in the literature, little effort has been invested in systematically replicating already published studies. In this regard, when talking about new technology, it is essential to acknowledge that technical progress evolves very quickly, and several of the reviewed studies were conducted more than a decade ago. Thus, their findings may have still-relevant but limited implications for recent technology (modern high-definition, more immersive VEs). As such, more studies should be performed based on current technology and devices. Furthermore, some studies should aim to replicate old findings using new devices. This line of research can help with the development of a more comprehensive theoretical framework for presence and related constructs.

Future research should also focus on the role of presence in shaping human performance. The role of presence in new media and its relationship with user performance may be of great interest for applied research in the field of IVTs. More empirical research is needed to understand better how the sense of presence (and related factors, e.g., emotional involvement) may represent a positive or a negative factor for human performance, depending on different uses of IVTs. The authors of the present review wish to stimulate the replication of the published scientific research on the physiological correlates of presence, especially in those cases where the published studies have reported conflicting or inconclusive results. At the same time, this review underlines the theoretical problems with the definition of presence as a psychological construct.

## Author Contributions

SG was responsible for retrieving and reviewing the current literature, writing the drafts of the article, including figures and tables, and revise all the previously submitted versions of the manuscript. KL provided funding for the project, extensively commented on the earlier drafts of the manuscript, and revised the manuscript at every stage of its development. Both authors agreed on and commented on the final version of the manuscript prior to submission.

### Conflict of Interest

The authors declare that the research was conducted in the absence of any commercial or financial relationships that could be construed as a potential conflict of interest.

## Figures and Tables

**Table:** 

**Table of Abbreviations**		
**Abbreviation**	**Explanation**	**Category**
IVT VE VR	Immersive Visual Technology Virtual Environment Virtual Reality	General
E2I IEQ IPQ ITC-SoPI ITQ MEC-SPQ MPS MRJPQ PQ SUS UCL	E^2^I Questionnaire (Lin et al., 2002) Immersive Experience Questionnaire (Jennett et al., 2008) Igroup Presence Questionnaire (Schubert et al., 2001) ITC-sense of presence inventory (Lessiter et al., 2001) Immersive Tendencies Questionnaire (Witmer and Singer, 1998) MEC Spatial Presence Questionnaire (Vorderer et al., 2004) Multimodal Presence Scale (Makransky et al., 2017a) Modified Reality Judgment and Presence Questionnaire (Anderson et al., 2017) Presence Questionnaire (Witmer and Singer, 1998). Slater-Usoh-Steed Questionnaire (Slater and Steed, 2000) University College of London Questionnaire (Usoh et al., 1999; Meehan, 2001)	Questionnaires
BOLD ECG/EKG EDA EEG EMG ERP(s) fMRI GSR HR PPG SC ST	Blood-oxygen-level-dependent (signal) Electrocardiography Electrodermal Activity Electroencephalography Electromyography Event-related potential(s) Functional Magnetic Resonance Imaging Galvanic Skin Responses Heart Rate Photoplethysmography Skin Conductance Skin Temperature	Physiology
